# Predictive Value of Optic Nerve Sheath Diameter for Diagnosis of Intracranial Hypertension in Children With Severe Brain Injury

**DOI:** 10.3389/fped.2022.894449

**Published:** 2022-06-06

**Authors:** Fleur Cour-Andlauer, Aurélie Portefaix, Isabelle Wroblewski, Muriel Rabilloud, Fabienne Bordet, Bérengère Cogniat, Capucine Didier, Robin Pouyau, Frédéric V. Valla, Behrouz Kassai-Koupai, Gaëlle Siméon, Tiphanie Ginhoux, Sonia Courtil-Teyssedre, Etienne Javouhey

**Affiliations:** ^1^Hospices Civils de Lyon, Paediatric Intensive Care Unit, Mother and Children University Hospital, Bron, France; ^2^EA 7426 “Pathophysiology of Injury-Induced Immunosuppression” (Université Claude Bernard Lyon 1 – Hospices Civils de Lyon – bioMérieux), Joint Research Unit HCL-bioMérieux, Lyon, France; ^3^Clinical Investigation Center CIC 1407, Hospices Civils de Lyon, Bron, France; ^4^CNRS, UMR 5558, Laboratory of Biometry and Evolutionary Biology, University of Lyon 1, Villeurbanne, France; ^5^Pediatric Intensive Care Unit, Grenoble Alpes University Hospital, Grenoble, France; ^6^Université de Lyon, Lyon, France; ^7^Université Lyon 1, Villeurbanne, France; ^8^Hospices Civils de Lyon, Pôle Santé Publique, Service de Biostatistique et Bioinformatique, Lyon, France; ^9^CNRS, UMR 5558, Laboratoire de Biométrie et Biologie Évolutive, Équipe Biostatistique-Santé, Villeurbanne, France; ^10^Department of Anesthesia and Intensive Care, Hospices Civils de Lyon, Mother and Children University Hospital, Bron, France; ^11^Pharmacology and Therapeutics Laboratory, University of Lyon 1, Lyon, France

**Keywords:** children, brain injury, intracranial hypertension, ultrasonography, optic nerve

## Abstract

**Background and Aims:**

Intracranial Hypertension (ICH) is a life-threatening complication of brain injury. The invasive measurement of intracranial pressure (ICP) remains the gold standard to diagnose ICH. Measurement of Optic Nerve Sheath Diameter (ONSD) using ultrasonography is a non-invasive method for detecting ICH. However, data on paediatric brain injury are scarce. The aim of the study was to determine the performance of the initial ONSD measurement to predict ICH occurring in children with severe brain injury and to describe the ONSD values in a control group.

**Methods:**

In this cross-sectional study, ONSD was measured in children aged 2 months-17 years old with invasive ICP monitoring: before placement of ICP probe and within the 60 min after, and then daily during 3 days. ONSD was also measured in a control group.

**Results:**

Ninety-nine patients were included, of whom 97 were analysed, with a median (IQR) age of 8.7 [2.3–13.6] years. The median (IQR) PIM 2 score was 6.6 [4.4–9.7] and the median (IQR) PELOD score was 21 [12–22]. Aetiologies of brain injury were trauma (*n* = 72), infection (*n* = 17) and stroke (*n* = 8). ICH occurred in 65 children. The median (IQR) ONSD was 5.58 mm [5.05–5.85]. ONSD performed poorly when it came to predicting ICH occurrence within the first 24 h (area under the curve, 0.58). There was no significant difference between the ONSD of children who presented with ICH within the first 24 h and the other children, with a median (IQR) of 5.6 mm [5.1–5.9] and 5.4 mm [4.9–5.8], respectively. Infants aged less than 2 years had a median (IQR) ONSD of 4.9 mm [4.5–5.2], significantly different from children aged more than 2 years, whose median ONSD was 5.6 mm [5.2–5.9]. Age, aetiology or ICP levels did not change the results. Thirty-one controls were included, with a median age of 3.7 (1.2–8.8) years. The median (IQR) of their ONSD measurement was 4.5 mm [4.1–4.8], significantly lower than the patient group.

**Conclusion:**

In a paediatric severe brain injury population, ONSD measurement could not predict the 24 h occurrence of ICH. Severity of patients, timing and conditions of measurements may possibly explain these results.

## Introduction

Intracranial hypertension (ICH) is a severe complication of paediatric acute brain disorders that can be life-threatening. Establishing a clinical diagnosis of ICH is, at times, difficult due to the lack of cooperation from infants, the lack of specificity of ICH clinical manifestations and because sedation is often necessary for disease management. To confirm a diagnosis of ICH, the invasive measurement of intracranial pressure (ICP) is performed using an intraparenchymal probe or intraventricular catheter. This invasive device remains the gold standard to diagnose ICH and to monitor ICP. Several non-invasive tools have been also developed to detect ICH, including the ultrasound measurement of the Optic Nerve Sheath Diameter (ONSD). The optic nerve is an extension of the central nervous system (CNS) surrounded by a sheath. This sheath, which is an extension of the dura mater and contains cerebrospinal fluid, can indeed distend depending on ICP changes (1–3).

There is an extensive amount of literature available regarding adult patients that shows a good correlation between ONSD and ICP measurement (4–6). However, no threshold measurement has currently met general approval. Aletreby et al. have recently found, in an adult meta-analysis, a cut-off of ONSD between 4.8 and 6.4 mm to detect ICH (7). However, in adults with severe trauma brain injury (TBI), another meta-analysis found a cut-off of 5.8 mm to predict ICH (8). In children, data are much scarcer, with results as well as sensitivity and specificity values varying greatly. A recent paediatric meta-analysis has reviewed the diagnostic performances of ONSD and found sensitivities between 39 and 100% and specificities between 22 and 100% (9). Some studies found a good correlation between ONSD and ICP, such as Kerscher (10) or Padayachy (11), whereas others do not find this correlation, such as Biggs (12) and Sharawat (13). It is noteworthy that few paediatric studies used invasive measurement of ICP (10, 11, 13, 14).

The main objective of the present study was to determine the performance of the ONSD measurement to predict ICH occurring within the first 24 h of placing an invasive ICP measurement probe in severe brain-injured children hospitalised in paediatric intensive care units (PICUs). The secondary objectives were to compare infants to children aged over 2 years old, to compare the ONSD measurement depending on the aetiology of brain injury, to describe the evolution of the ONSD measurement over a period of 3 days and to describe the ONSD values in sedated, ventilated children without brain injury (control group). We hypothesised that ONSD measurement would identify ICH in severe brain-injured children with sufficient precision.

## Materials and Methods

### Setting and Patients

This prospective, cross-sectional and multicentre study was conducted in the Lyon and Grenoble PICUs, in France. We included consecutively children aged from 2 months to 17 years old, hospitalised for severe brain injury requiring invasive ICP monitoring according to the physician in charge of the patient. Children with a history of ocular disease, brain malformation or brain tumour, or chronic ICH were not included.

Moreover, we included a control group of children of the same age range who were sedated, intubated-ventilated and who did not have any brain injury. Before the inclusion of a child, an informed consent form was signed by both parents. A favourable opinion from the Comité de Protection des Personnes (CPP) Sud Est II (the research ethics committee for South-East of France) was granted on January 5, 2011. An authorisation from the AFSSAPS (former French National Agency for Medicines and Health Products Safety) was granted on December 20, 2010. The study was reported on clinicaltrials.gov (NCT01796015) and funded by the invitation to tender Actions Incitatives HCL 2009 N° D50705. Promotion was performed by the Hospices Civils de Lyon (HCL - n°2010-A00890-39).

In the PICUs participating in the study, invasive ICP monitoring is commonly performed (Camino, Integra LifeSciences, San Diego, CA, United States) for severe acute brain injury, such as severe TBI, CNS infection with coma, or severe stroke as suggested by some national or international guidelines (15–18).

### Design and Data Collection

Optic Nerve Sheath Diameter measurement by ultrasonography is well defined and standardised (19): the diameter is measured 3 mm behind the eyeball, using the electronic calliper of the ultrasonography machine along a two-dimensional axis perpendicular to the optic nerve, with a linear probe. For each optic nerve, one sagittal measurement and one transversal measurement are taken. The final measurement constitutes the mean of the four measurements taken during each examination. For the patient group, the first examination was performed within the 15 min prior to ICP probe placement. A second examination was performed within the 60 min after ICP probe placement. The examinations were then repeated once a day for 3 days, and performed long before or after any care procedure that may alter ICP. For the control group, a single ultrasound examination was performed.

All the examinations were performed by practitioners trained in this procedure. The ultrasound machines used were the HD11XE (Philips Medical Systems, Bothell, WA, United States) with a 7.5 MHz linear probe in Lyon and the Vivid S6 (GE Healthcare, Milwaukee, WI, United States) with a 9 MHz linear probe in Grenoble. All the examinations were recorded and systematically reread by a first reader (F.C.A. or E.J. if F.C.A. performed the recorded examination), blinded from the ICP value, after all inclusions. If the difference between the reread value and the value initially collected by the practitioner was higher than 0.6 mm, a second rereading between both readers, blinded from the ICP value and the measurement of the first reader, was performed to reach a consensus. The measurement used for the statistical analysis was the one found during rereading. The ICH definition was based on the ICP values with a 15-mmHg threshold for infants aged less than 2 years and a 20-mmHg threshold for children aged over 2 years old. These thresholds were chosen because they were used in clinical practice in both participating centres and because physiological values of ICP are lower in infants compared to older children (15).

Clinical data were collected in a prospective way: demographical data (age, genre, weight), aetiologies of the brain disorders (trauma, CNS infection, stroke) and severity scores (Paediatric Index of Mortality score [PIM-2] at admission and Paediatric Logistic Organ Dysfunction [PELOD] 24 h after admission).

### Statistical Analysis

The accuracy of ONSD for the diagnosis of ICH was quantified by the Area Under the ROC Curve (AUC).

A sample size of 97 patients was determined in order to estimate the AUC with a precision of 7% for a 95% confidence interval (CI), under the assumption of an ICH prevalence equal to 50% in the studied population and for an expected AUC of 91%.

The empirical ROC curves were built using the ONSD measured within the 15 min prior to ICP probe placement. The ICH status was determined using the ICP measured during the first 24 h following the ICP probe placement. The AUC estimate and the 95% CI were obtained using the non-parametric method of Delong.

The sensitivity and specificity of ONSD were estimated using the positivity thresholds defined by Padayachy (20). The 95% CI was built using the method of Wilson.

The overall intra-patient correlation between ONSD and ICP measured together several times during the follow-up was estimated using the method allowing to take into account the repeated measures. The non-parametric test of Mann and Whitney was used to compare quantitative variables. The Chi-square test or the Fisher exact test were used to compare qualitative variables.

All the analyses were carried out using the statistical software R, version 3.6.3. The ROC curves and the AUC estimates were obtained using the package pROC (21).

## Results

**FIGURE 1:**
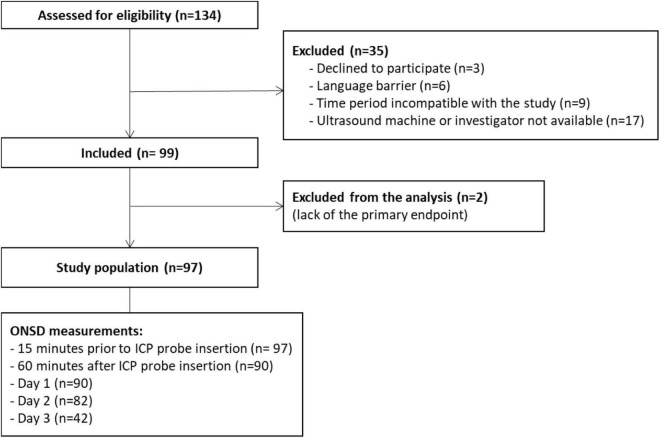
Patients’ flow diagram. ONSD, Optic Nerve Sheath Diameter; ICP, intracranial pressure.

**FIGURE 2:**
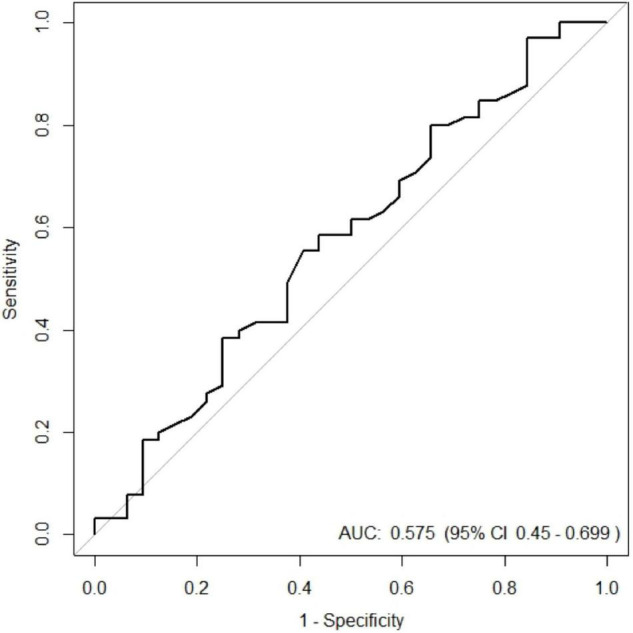
Empirical ROC curve of Optic Nerve Sheath Diameter (ONSD) used to diagnose an intracranial hypertension occurring within the first 24 h following the measurement of ONSD in a paediatric brain injury population. AUC, area under the curve.

**FIGURE 3:**
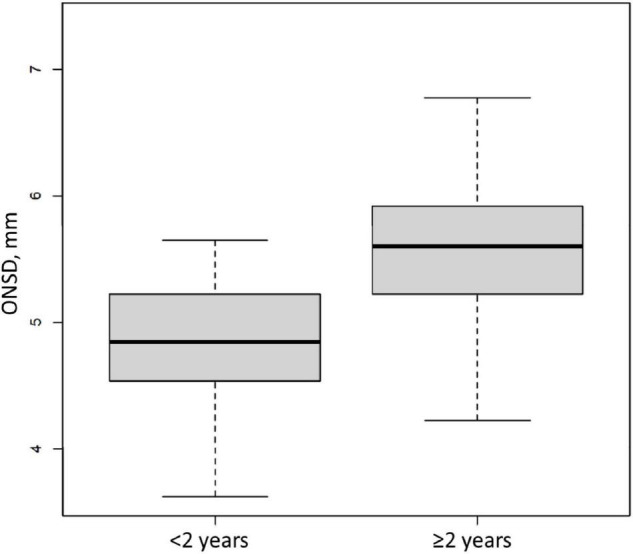
Initial Optic Nerve Sheath Diameter (ONSD) in a paediatric brain injury population according to the age. Box and whisker plots represent interquartile range and 95% CI, respectively, and the horizontal bar indicates the median, *p* < 0.001.

**FIGURE 4:**
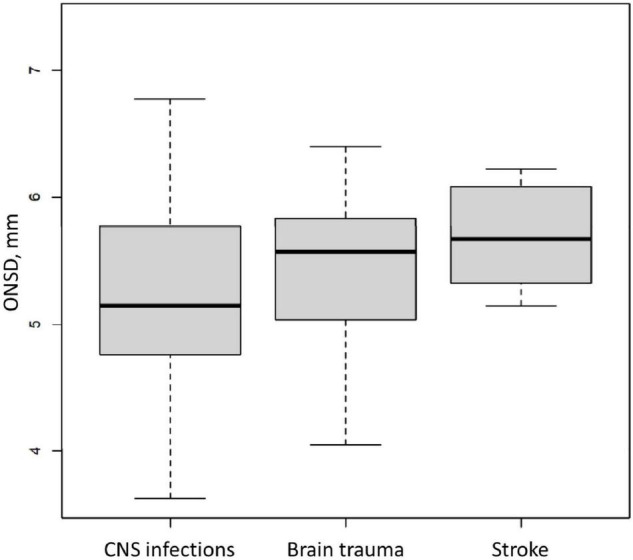
Initial Optic Nerve Sheath Diameter (ONSD) in a paediatric brain injury population according to the aetiology. CNS, central nervous system. Box and whisker plots represent interquartile range and 95% CI, respectively, and the horizontal bar indicates the median.

**FIGURE 5:**
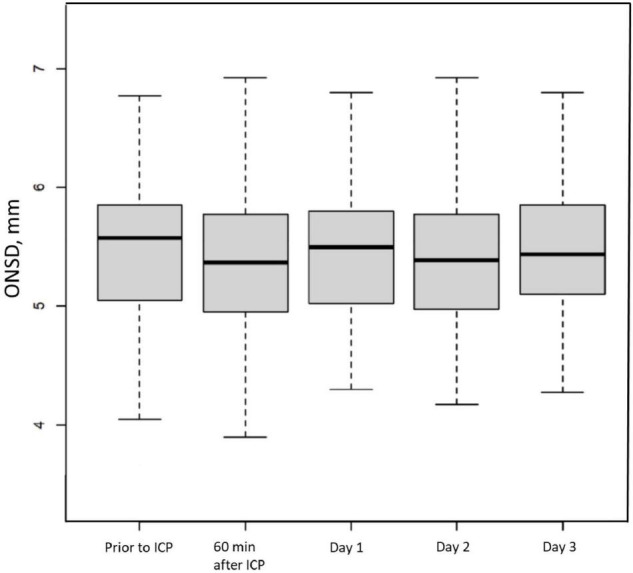
Time course of Optic Nerve Sheath Diameter (ONSD) in a paediatric brain injury population. Box and whisker plots represent interquartile range and 95% CI, respectively, and the horizontal bar indicates the median. ICP, intracranial pressure.

Between April 2011 and February 2017, 99 patients were included (Figure 1), 97 of whom were retained for the statistical analysis. Patients’ characteristics are described in Table 1. During the study period, 404 measurements were performed and 34 examinations were not recorded, which means that rereading was not feasible. Therefore, 269 measurements were reread once and 101 needed a second rereading for consensus. The median (interquartile range [IQR]) of the time interval between the first ONSD measurement and the ICP probe placement was 15 min [10–30]. The median (IQR) of the ICP measurement at baseline was 18.5 mmHg [10.7–28]. The median (IQR) of the ONSD measurement before placing the ICP probe was 5.58 mm [5.05–5.85]. The overall intra-patient correlation between ONSD and ICP was estimated at 0.07 (95% CI −0.07–0.21). ICH occurred in 65 children (67%) within the first 24 h. There was no significant difference between the ONSD of children who presented with ICH within the first 24 h and the other children, with a median (IQR) of 5.6 mm [5.1–5.9] and 5.4 mm [4.9–5.8], respectively (*p* = 0.42). The AUC was estimated at 58% (95% CI 45–70%) and the ROC curve is presented in Figure 2. Infants aged less than 2 years (*n* = 20) had a median ONSD (IQR) of 4.9 mm [4.5–5.2], significantly different from children aged more than 2 years (*n* = 77), whose median ONSD was 5.6 mm [5.2–5.9] (*p* < 0.001) (Figure 3). Based on the thresholds proposed by Padayachy (20), the diagnostic performance of ONSD at the 5.16 mm threshold in infants aged less than 1 year and at the 5.5 mm threshold in older children showed a 60% sensitivity (95% CI: 48–71%) and a 53% specificity (95% CI: 36–69%). Moreover, we performed a subgroup analysis depending on the age of the children and the aetiology of the brain disorders. ONSD did not differ according to the aetiology (CNS infection, severe TBI or stroke) (Figure 4). The diagnostic performance of ONSD to predict the occurrence of ICH was similar in children aged less or more than 2 years with an AUC (95% CI) of 52% (22–82%) and 58% (43–72%), respectively, and comparable whatever the aetiology with an AUC (95% CI) of 65% (34–97%) in children presenting with a CNS infection and of 54% (39–68%) in children with severe TBI. Finally, the distribution of ONSD was very similar regardless of the measurement time during the 4 days of the study (Figure 5).

**TABLE 1:** Patient and control characteristics.

Variable	Overall patient population (*n* = 97)	With ICH (*n* = 65)	Without ICH (*n* = 32)	Control group (*n* = 31)
**Demographics**				
Age, year, median (IQR)	8.7 (2.3–13.6)	8.6 (2.4–13.1)	9.0 (2.1–14.6)	3.7 (1.2–8.9)
Male, n (%)	63 (64.9)	42 (64.6)	21 (65.6)	19 (61.3)
Weight, kg, median (IQR)	25 (15–50)	25 (15.5–49)	25.2 (14.5–52)	15.5 (10–30) ^†^
Height, cm, median (IQR)	127 (93–156)	128 (98.5–155)	126 (89.5–159)	101 (75.5–131)
**Severity scores**				
PIM2, median (IQR)	6.6 (4.4–9.7)	7.1 (4.4–11.6)	6.5 (4.3–8.4)	–
PELOD, median (IQR)	21 (12–22)	21 (12–22)	17 (12–21.3)	–
**Reason for ICP monitoring**				
CNS infection, n (%)	17 (17.5)	13 (20)	4 (12.5)	–
Traumatic brain injury, n (%)	72 (74.2)	45 (69.2)	27 (84.4)	–
Stroke, n (%)	8 (8.2)	7 (10.8)	1 (3.1)	–
ONSD*, median (IQR)	5.58 (5.05–5.85)	5.58 (5.10–5.88)	5.36 (4.88–5.76)	4.51 (4.11–4.83) ^†^
**Mortality**, n (%)	8 (8.2)	7 (10.8)	1 (3.1)	0 (0)

*For patients, within the 15 min prior to the placement of the ICP probe. Dashes indicate variables only reported for patients. ^†^Indicate significantly results between control and overall patient population. ICH, intracranial hypertension; PIM2, Paediatric Index of Mortality 2; PELOD, Paediatric Logistic Organ Dysfunction; ICP, intracranial pressure; CNS, central nervous system.

**FIGURE 6:**
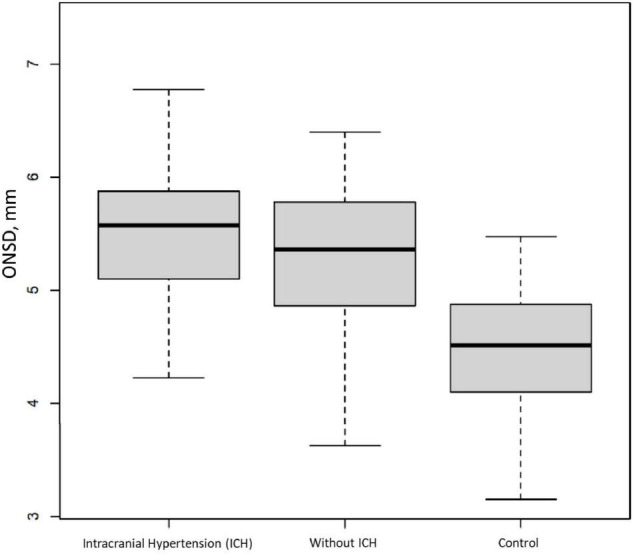
Initial Optic Nerve Sheath Diameter (ONSD) in a paediatric brain injury population. Comparison between patients with or without Intracranial Hypertension (ICH) and the control group. Box and whisker plots represent interquartile range and 95% CI, respectively, and the horizontal bar indicates the median.

Between April 2011 and March 2015, 31 children were included in the control group. Their demographic characteristics are described in Table 1. The median (IQR) of their ONSD measurement was 4.5 mm [4.1–4.8], significantly lower than the patient group (*p* < 0.0001) (Figure 6). In this control group, the ONSD in children aged more than 2 years was larger than the ONSD in children aged less than 2 years, with a median (IQR) of 4.6 mm [4.4–4.9] and 4.1 mm [4.0–4.5], respectively.

All examinations were performed uneventfully.

## Discussion

In this multicentre study including PICU children, the ONSD was not able to predict ICH occurring within 24 h of ICP probe placement. These data are concordant with those described by Biggs et al. (12), even if their population seemed to be less severely affected with a prevalence of ICH in only 6% of their patients, compared to 67% in our study. A paediatric meta-analysis performed by Bhargava et al. (9) concluded on the inability to identify a threshold for ONSD to predict ICH, with pooled sensitivity of 93% but specificity of only 74%. Our results, however, diverge from those described by other authors in paediatrics. Kerscher et al. (10) found a good correlation between ONSD and ICP in neurosurgical patients, of whom 7% were in ICUs. Our patients were all in ICUs in more severe and acute conditions. This element may modify the ONSD physiology and distensibility. Padayachy et al. also found a good correlation between ONSD and ICP at the time of a neurosurgical procedure (11, 20). Their patients were included during surgical procedure with an ICH prevalence of 42%. Moreover, aetiologies varied from our population. They included 17% of traumatic brain injury versus 74% in our population, and they did not include CNS infection. These points could lead to differences in ICH mechanism and ONSD physiology, which may explain various results.

The ONSD values were significantly higher in patients compared to those of controls. This result was already found in a usual way, both in adults (8, 19, 22, 23) and in children (1, 13, 24–26).

In the patient group, as in the control group, children under 2 years of age have an ONSD significantly smaller compared to children over 2 years of age. There is indeed a growth in the optic nerve sheath, described by Fontanel et al. (25), found up until 10 years of age, but it is particularly pronounced during the first year of life (27). In our study, the children in the control group were younger (median age of 3.7 years) than the patient group (median age of 8.7 years), which may lead to a bias in the interpretation of our results. Fontanel et al. nevertheless described a very moderate growth in this age group (25). It is therefore conceivable that our results may well be a consequence of the neurological condition of our population, rather than related solely to age. However, our results obtained concerning variation with age suggest that age should be taken into account in the analysis of measurements, and that patients included in studies on ONSD should be stratified according to age.

In our patients, the ONSD results were comparable regardless of the cause of the brain injury. This analysis is hardly found in the literature. To our knowledge, only Padayachy et al. described different results depending on aetiology, with higher ONSD values in tumour or craniosynostosis patients (11). In these conditions, the mechanism of ICH is much slower than the causes of brain injury in our patients with different pathophysiological mechanisms that can alter the dynamics of evolution of ONSD.

Moreover, we did not observe a difference in ONSD over a period of 3 days in our patients with enlarged ONSD. We found enlargement at the outset without return to normal at a distance from acute ICH. In our study, the ONSD measurement therefore does not appear to be a relevant tool for monitoring our severe brain-injured patients. This result contradicts what has been observed by other authors. In other adult studies, changes in ICP led to correlated changes in ONSD in real time (28–30). In paediatrics, Vijay et al. also found rapid changes in ONSD in a very specific population having suffered from acute liver failure during acute episodes of ICH, with even a prognostic effect if the return to the baseline value occurred within 2 h (24). On the contrary, other authors found that changes in ICP were poorly correlated with changes in ONSD (10, 31).

The variability of results in the abundant literature on the subject could be explained by effects on the elasticity of the optic nerve sheath, as already described by Padayachy et al. (11), for which physiological studies would be useless. Stevens has recently taken an interest in the many methodological differences in ONSD measurement, which may explain the significant differences in our results, and has suggested four recommendations to follow in order to standardise the measurements in future studies (32). Although our measurements were collected after the publication of Steven’s article, they matched three of the four recommendations, i.e., to measure outside the hyperechoic band if visible, to use the papilla as the starting point of the 3-mm depth and to use a high-frequency linear probe. The last recommendation suggests a mechanical index smaller than or equal to 0.3. We do not possess this information *a posteriori*.

The patients included in our study were particularly severely affected, as shown by their high severity scores, with a median PIM2 score of 6.6 and a PELOD score of 21, and the occurrence of ICH in 67% of them. This severity appears to be more significant than that of patients in the other paediatric studies (10, 12, 33). This point may explain part of our results. Indeed, the neurological severity of patients included in the study resulted in an active therapeutic management aiming to decrease ICP, even before ICP probe placement. In the group of patients without ICH, some of them may have presented with ICH, but it was treated and controlled from the start. Therefore, it seems to us that the ONSD measurement does not appear to be a relevant tool for discriminating severe brain-injured patients from the outset. Other authors such as Aletreby et al. (7) and Kerscher et al. (10) also suggested that ONSD may be a complementary tool in emergency departments rather than in ICUs, without replacing invasive measurements.

Regarding the blind rereading of all examinations, the methodology used was rigorous and systematic, allowing us to give value to our results. Even if one of the readers was able to perform the measurement themselves, rereading was performed after all inclusions, which were spread over more than 6 years, allowing concomitant ICP to be forgotten. In this way, the process of blinding was respected.

Our study has nevertheless some limitations. Although the number of inclusion was consistent with the calculation of the number of subjects needed to answer the primary objective, the number of patients per subgroup, and particularly for children younger than 2 years, is limited. As with any ultrasound examination, there is inter-rater variability. The training of the investigators was, however, standardised and they should have performed a minimum of 20 supervised ultrasound examinations in order to limit variability as much as possible. Any rereading of ultrasound examination remains a static measurement of an examination that is, by definition, dynamic thus resulting in inaccuracies. We conducted the analyses of the primary endpoint by taking into account the measurement collected by the investigators (data not shown). The results were similar. Finally, there is no general consensus on the definition of ICH in the paediatric population. The thresholds chosen in the study are, however, accepted by several teams (34) and already used in the literature concerning ONSD (10, 11, 20).

## Conclusion

The ONSD measurement is not associated with ICH occurring within 24 h in a paediatric population with severe brain injury. Brain injury results in an increased ONSD, which continues at least 3 days and is present at any age. The thresholds predicting ICH may probably be adjusted to the age of the patients. Further studies using rigorous measurement methodology are still needed to target the population for whom this feasible, inexpensive and rapid examination may be useful.

## Data Availability Statement

The original contributions presented in the study are included in the article/supplementary material, further inquiries can be directed to the corresponding author.

## Ethics Statement

The studies involving human participants were reviewed and approved by the Comité de Protection des Personnes (CPP) Sud Est II (the Research Ethics Committee for South-East of France). Written informed consent to participate in this study was provided by the participants’ legal guardian/next of kin.

## Author Contributions

FC-A, AP, BK-K, TG, and EJ involved in the study design, data collection, and data interpretation. IW, FB, BC, CD, RP, FV, and SC-T involved in the data collection. MR performed the statistical analysis. FC-A wrote the first draft of the manuscript. AP, EJ, TG, and BK-K contributed to interpretation of the data and revising the manuscript. GS revised the manuscript. All authors contributed to manuscript revision, read, and approved the submitted version.

## Conflict of Interest

The authors declare that the research was conducted in the absence of any commercial or financial relationships that could be construed as a potential conflict of interest.

## Publisher’s Note

All claims expressed in this article are solely those of the authors and do not necessarily represent those of their affiliated organizations, or those of the publisher, the editors and the reviewers. Any product that may be evaluated in this article, or claim that may be made by its manufacturer, is not guaranteed or endorsed by the publisher.
